# *Cutibacterium acnes* in breast implants: an underestimated bacterial infection and review of the literature

**DOI:** 10.1093/jscr/rjad042

**Published:** 2023-02-14

**Authors:** Sam Hanna, Shauna Manuel, Jenalle Baker, Jason Diab, Zackariah Clement

**Affiliations:** Tweed Hospital, NSW, Australia; John Flynn Private Hospital, QLD, Australia; University of New South Wales, School of Medicine, Sydney, Australia; Griffith University, Queensland, Australia; Griffith University, Queensland, Australia; Tweed Hospital, NSW, Australia; John Flynn Private Hospital, QLD, Australia; University of New South Wales, School of Medicine, Sydney, Australia; University of Notre Dame, School of Medicine, Sydney, Australia; Tweed Hospital, NSW, Australia; John Flynn Private Hospital, QLD, Australia

## Abstract

The role of bacteria and breast implant illness is an emerging area of interest for surgeons and clinicians. The most common cause of surgical readmission remains post-operative infectious complications. *Cutibacterium acnes* is an anaerobic, gram-positive organism that is part of the normal human microbiota. In certain circumstances, it may cause chronic infections and capsular contractures in breast implant-related complications. This case series outlines patients with bilateral capsular contractures and growth of *C. acnes*. The patients were managed surgically with the removal of bilateral breast implants with en bloc capsulectomy and oral antibiotics without complications. This report will outline the pathology of *C. acnes*, association with breast implant-associated anaplastic large cell lymphoma and review of the literature.

## INTRODUCTION

Breast augmentation is one of the most popular cosmetic procedures with a global estimate over 1.6 million procedures performed annually [1]. In Australia, ~20 000 women undergo this procedure annually with 75% for cosmetic augmentation and 25% for reconstruction [2]. Breast implant complications can present with a wide range of symptoms, collectively referred to as breast implant illness (BII); these include fatigue, chronic pain, rash, body odour, irregular heart rate, anxiety, neurologic abnormalities, hair loss and endocrine dysfunction [3]. The relationship between bacteria growth and BII is emerging in the literature with greater attention because of its prevalence as more patients present with BII and Autoimmune Syndrome Induced by Adjuvants after Silicone Breast Augmentation Surgery. *Cutibacterium acnes* is an under-recognised, gram-positive anaerobe that can be part of the normal microflora. However, in certain circumstances it can present as a causative organism of breast augmentation surgery in the form of capsular contractures. This article will outline the pathology and relationship of *C. acnes* with breast surgery and review of the literature.

## CASE SERIES

A retrospective review was conducted from two institutions between January 2012 and July 2022. The inclusion criteria were adult patients above the age of 18 years with confirmed radiological and histopathological findings of *C. acnes* following breast implants. Patient consent was obtained in keeping with local ethical clinical practice and guidelines. Data collected included basic demographics, implant history and symptoms, time to presentation, imaging and management. All patients underwent base line bloods, ultrasound (US) and magnetic resonance imaging (MRI).

We report a total of three patients who presented with mastalgia and breast swelling following breast implants with histopathological confirmation of *C. acnes*. The mean age was 32 (±7.5) years. The average implant age was 8 (±1.8) years ranging from 6 to 10 years. The average onset of symptoms before presentation to a surgeon was 18 months (±21) ranging from 1.5 to 48 months. The common symptoms included breast swelling and mastalgia. All patients had bilateral capsulectomy with the removal of implants (Fig. 2). All patients had resolution of symptoms with a minimum of 1-year follow-up without complications.

### Case 1

A 27-year-old nulliparous female presented to the emergency department with a 6-week history of left breast swelling, hardness and mastalgia. Her past medical history included endometriosis, polycystic ovarian syndrome, migraine, anxiety, depression and regional lower limb pain syndrome. She underwent breast augmentation with silicone textured breast implants a decade ago. Clinically, her vital signs were stable with no haemodynamic instability or features of infection. Examination revealed bilateral breast asymmetry with both breasts extremely hard and tender and the left was worse than the right. There were bilateral prominent axillary nodes. Her baseline biochemical panel was normal.

**Figure 1:**
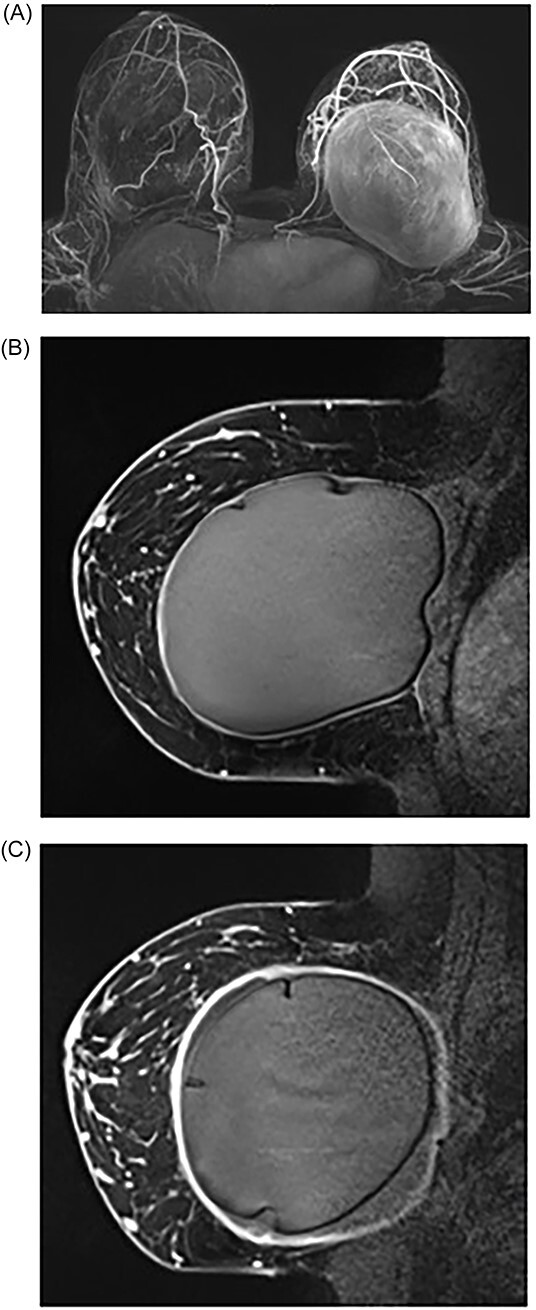
MRI of breasts showing left capsular lesion. (**A**) T1-weighted delayed subtraction axial MRI of breasts demonstrating intracapsular rupture with scattered foci of signal abnormalities consistent with disruptions in the silicone-matrix. (**B**) T1-weighted contrast sagittal MRI of right breast demonstrating mild diffuse thickening of the fibrous capsule with no nodularity and no abnormal enhancement. (**C**) T1-weighted contrast sagittal MRI of left breast demonstrating intracapsular rupture. The implant shell demonstrates several folds and indurations with no gross disruption

She underwent breast MRI, which showed left breast intracapsular rupture, moderate volume peri-implant fluid and diffuse thickening with avid enhancement of the fibrous capsule (Fig. 1). An US-guided left peri-implant fluid aspiration returned CD30 negative with no pathological features of breast implant-associated anaplastic large cell lymphoma (BIA-ALCL). She underwent the removal of bilateral breast implants with en bloc capsulectomy. Intraoperative findings included bilateral double capsules and outer capsules densely adhered to the ribs, muscles and breast. The inner capsules were severely contracted and significantly adherent to the underlying textured implants with bilateral thick peri implant intracapsular fluid. Both implants were removed with double capsules with subsequently no silicone or fluid spillage.

Histopathological assessment showed a fibrous breast capsule with synovial metaplasia, focal refractile silicone-like material and patch mild chronic inflammation. There was no atypical lymphoid infiltrate present and no capsular/traumatic neuroma was seen. There was no active inflammation or evidence of neoplasia. Cultures from both capsules grew *C. acnes*. The patient was discharged with a 5-day course of oral cephalexin. Seroma from both breasts was aspirated and cultures grew *Cutibacterium avidum* and within 4 weeks there was no drainable seroma or clinical features of infection.

### Case 2

A 27-year-old female presented to her general practitioner with a 3-year history of bilateral mastalgia with worsening pain over the last 5 months. Her past medical history included tuberous breast asymmetry and asthma with no regular medications or allergies. She had no significant family or cancer history. She underwent breast augmentation surgery with textured silicone breast implants 6 years prior. On examination, both breasts demonstrated tender palpable implants and rippling with features of bilateral Grade 3 capsular contractures. There was no associated lymphadenopathy.

She underwent an US, which demonstrated intact implants and no evidence of suspicious lesions or axillary lymphadenopathy. An MRI was arranged, but because of severe breast pain, the patient was unable to tolerate it. The patient underwent the removal of bilateral breast implants with an en bloc capsulectomy. Intraoperative findings revealed that both implant capsules significantly adhered to the ribs, muscles and implants contained thick peri-implant fluid bilaterally.

Histopathological analysis showed a fibrous breast capsule with synovial metaplasia and focal refractile silicone-like material with associated foreign body-type multi-nucleated giant cells. There was no atypical lymphoid infiltrate present with no active inflammation or evidence of neoplasia. Cultures from the left capsule grew left *C. acnes*.

### Case 3

A 43-year-old female presented to her general practitioner with a 4-year history of worsening bilateral mastalgia. She also experienced multiple BII symptoms such as brain fog, lethargy, low mood, altered gut functions, food intolerance, bloating, loose stools, low libido. She had no other relevant past medical history and no family history of cancer.

She has cosmetic breast augmentation surgery 10 years ago. On examination, both breasts demonstrated general discomfort with bilateral Grade 2 capsular contracture and prominent bilateral axillary lymph nodes.

**Figure 2:**
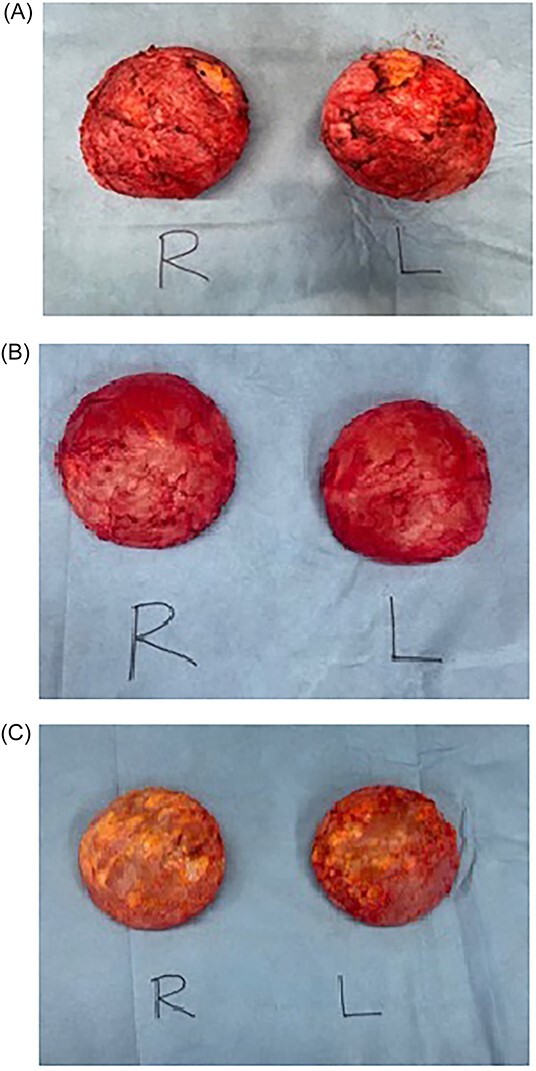
Intraoperative photos of bilateral breast implants. (**A**) Patient 1: right and left breast implant capsules. (**B**) Patient 2: right and left breast implant capsules. (**C**) Patient 3: right and left breast implant capsules

Breast MRI demonstrated disruptions in the silicone-matrix of both breast implants. There was no abnormality of concern to suggest BIA-ALCL and no features of breast carcinoma or adenopathy. The patient underwent the removal of bilateral breast implants with an en bloc capsulectomy and bilateral corrective mastopexy. Intraoperative findings revealed that both implant capsules significantly adhered to the ribs, muscles and implants contained thick peri-implant fluid bilaterally suspicious for a gel bleed (Fig. 2). There was significant peri-implant fibrotic scar tissue and adhesions with no extra-capsular siliconoma or mass seen with no features of ALCL. Histopathological analysis demonstrated a benign fibrous capsule with synovial metaplasia with no atypical lymphoid infiltrate present; cultures from the both capsules grew *C. acnes.*

## DISCUSSION

BII occurs in 1.1–2.5% of patients after aesthetic breast augmentation and up to 35% after breast implant reconstruction following mastectomy [4]. A systematic review reported one of the most common complications after breast augmentation surgery is capsular contracture with incidences up to 30% [5, 6]. The pathogenesis of capsular contracture is thought to be a result of a multifocal inflammatory process resulting in an excessive fibrotic reaction causing deformation and irritation of the breast [7]. The associated risk factors include indication for surgery (reconstructive versus cosmetic augmentations), history of pre- and post-operative radiation therapy, type of prosthesis used (texture and material) and positioning of the implant (subglandular versus submuscular) [6].

*Cutibacterium acnes* (formerly *Propionibacterium acnes*) is a slow-growing, gram positive human skin commensal that prefers anaerobic growth conditions. It is commonly involved in the pathogenesis of acnes [8], but also recognised as a pathogen in foreign body infection such as endocarditis, bone and prosthesis infections [9]. In a study of 139 capsulectomies, *C. acnes* was isolated in approximately half the positive cultures [10]. *Cutibacterium acnes* has many virulence strategies with differing growth potential in human tissue attributing to timely delay in clinical presentation [11, 12]. It can be problematic to manage because of its ability to produce biofilms on surgical material contributing to the pathogenesis of capsular contractures [13–16]. In our series, *C. acnes* was grown from cultures originating from the implant capsule, but Pittet’s systematic review suggests that the most likely origin of the pathogen is either the patient’s skin or colonised mammary ducts at the incision site [17].

A search of the literature was conducted using the terms ‘Propionibacterium or Cutibacterium acnes’ and ‘breast implants’, and ‘breast implant illness’ across multiple databases including Embase, PubMed and Google Scholar. Articles included were those that specifically measured systemic symptoms attributed to the breast implants and reported microbiology for the implant samples for *C. acnes* or *P. acnes.* Of 733 articles, six articles satisfied reported an average age of 43.8 years (±6.3). The literature reports 556 women experiencing systemic or extra-mammary symptoms of BII, ranged from fatigue, headaches, muscle cramps, loss of concentration and anxiety; <200 women reported mammary symptoms associated with BII. All cases were managed with explantation surgery either with or without capsulectomies. There were no cases of malignancy of neoplastic changes. Of 556 women, 106 patients (19%) grew *C. acnes* (Table 1). In our series patients had breast implants for 8.0 (±1.8) years with the average onset of symptoms before presentation was 18 months (±21).

**Table 1:** A literature review of case reports on *C. acnes* and BII

**Author**	**Year**	**Study design**	**Patient presentation/s**	**Surgical management**	**Histology**	**Microbiology**	**Results**
McCarthy *et al*. [24]	2022	Case report	A 38-year-old Caucasian, nonobese woman had undergone an elective subfascial implantation for hypomastia. Three weeks post-implantation, patient began to develop the following symptoms: constant dull headache, ‘brain fog’, psychological symptoms (sever anxiety and panic attacks) and hair loss.	Explanation surgery 8 months from initial placement.	Showed focal stromal fibrosis. No malignancy or neoplastic change.	Thio broth culture positive for *C. acnes*	Symptoms resolved within 1 week of explanation.
Metzinger *et al*. [25]	2022	Retrospective cohort study	In all, 200 women with self-reported symptoms consistent with BII opting for the removal of implants. BII symptoms included both mammary (mastodynia and capsular contracture) and extramammary symptoms (fatigue, loss of concentration, arthralgia, myalgia, rashes, ocular complaints, memory loss and autoimmune diagnosis). Average age of patients was 45.5 years (range: 29–73). Average range of implant duration unreported.	Total capsulectomy and breast implant removal.	No cases of BIA-ALCL.	68.5% of patients had at least one positive bacterial culture. Most common organism (49.6%) was *P acnes.*	96% of patients reported significant decrease in symptoms post-surgery.
McGuire *et al*. [26]	2022	Prospective cohort study	Two groups of women undergoing breast implant removal plus a control group: (i) 50 women with systemic symptoms of BII (average age 44.5 years), (ii) 50 women with no symptoms of BII (average age 46.9 years) and (iii) 50 controls with no implants undergoing mastopexy (average age 46.5 years). Implants of unclear duration. Symptoms of BII were not specifically reported.	Explantation surgery with either partial or total capsulectomies.	Both groups showed fibrosis, lymphoplasmacytic infiltration, calcifications, giant cells, foam cells and epithelioid histiocytic capsules within the removed tissue.	48% of the group with BII and 46% of the group without BII had positive cultures. *Cutibacterium acnes* was the most commonly isolated bacteria identified. 44% of the BII group and 32% of the other group had *C. acnes,* although this difference was not statistically significant.	Symptom improvement at 3–6 weeks and at 6 months was not significantly associated with the presence of *C. acnes* in either those with BII symptoms or those without.
Katsnelson *et al*. [27]	2021	Retrospective cohort study	In all, 248 women presenting with systemic symptoms of BII and subsequently underwent bilateral implant removal. Average age at presentation for removal was 45 years (range 22–72 years), average age at implantation was 29.5 years. Symptoms of BII included generalised pain, fatigue, brain fog, migraines, headaches, anxiety, arthritis, vision changes, dyspnoea, hair loss, weight gain, back pain, rashes, gastrointestinal issues and depression.	Explantation with or without capsulectomy, either partial or total.	Acute or chronic inflammation was evident in 23% of the capsules. One capsule showed atypical lymphocytic infiltration, which was CD30 negative when testing for BIA-ALCL.	Overall, 14 patients had positive cultures. *Propionibacterium* sp. unspecified was identified in one patient. *Cutibacterium acnes* was identified in one patient.	Only 46 patients had recording of BII symptoms at follow-up, with 44 of these patients reporting a decrease in symptoms post-surgery.
Lee *et al*. [28]	2020	Prospective cohort study	In all, 50 women presenting with self-diagnosed BII and requesting explantations (mean age: 42 years), compared with matched control cohort having no BII and removal with replacement of implants (mean age: 46 years). Duration of implants were the same for each group (3–5 years). Symptoms of BII included fatigue, arthralgia, brain fog, myalgia, memory loss, difficulty concentrating, autoimmune conditions, rash and visual disturbances/dry eyes.	Bilateral capsulectomies with en bloc removal of implant and overlying capsule where possible.	BII group had significantly higher (50%) rate of synoviocyte metaplasia compared with controls.	BII group had a 6-fold greater rate of positive cultures. *Propionibacterium acnes* represented the majority of cases (12/18) in the BII group. Only three of the control group had positive cultures, none with *P. acnes*	78% of the BII reported significant resolution in their preoperative BII symptoms during the follow-up period. There was no statistically significant difference in resolution of symptoms between those with *P. acnes* compared with those with negative cultures.
Dowden [29]	1994	Series of case reports	**Case 1:** 38-year-old woman experiencing seromas, extrusion (requiring several replacements), breast discomfort, muscle aches, fatigue, diarrhoea. Implants for ~5 years. **Case 2:** 50-year-old woman, breast cancer reconstruction, with implants inserted for unclear duration of years. Experiencing firmness and chest-wall discomfort, discomfort in the legs, shoulders, neck, left hip, muscle aches. **Case 3:** 37-year-old woman with unilateral breast implant of 12-year duration with no problems. Began experiencing severe fatigue, photophobia, subsequently diagnosed with chronic fatigue syndrome. **Case 4:** 41-year-old woman with breast implants for a duration of 15 years, with symptoms of illness for 10 years. Symptoms included chronic fatigue, being unable to remain awake past early evening. **Case 5:** 38-year-old woman with breast implants of 7-year duration experiencing symptoms of illness from the first year onwards. Symptoms included diarrhoea, severe fatigue, insomnia, irregular periods, hot flashes. All blood tests were normal. **Case 6:** 28-year-old woman with breast implants of 11-year duration, experiencing symptoms beginning ~3 years after implantation. Symptoms included fatigue, pain in feet, hands and hips. **Case 7:** 54-year-old woman with implants of 3-year duration experiencing illness symptoms from insertion. Symptoms included burning discomfort in chest and shoulders, fatigue and memory impairment.	Explantation with or without partial capsulectomy	NA	Cultures were taken either by irrigation or swab. **Case 1:** cultures grew *P. acnes* **Case 2:** nil growth **Case 3:** nil growth **Case 4:** cultures grew *Staphylococcus epidermidis* **Case 5**: cultures grew *S. epidermidis* and *P. acnes* **Case 6**: cultures grew *S. epidermidis* **Case 7:** cultures grew *S. epidermidis* and *P. acnes*	**Case 1:** fatigue and muscle aches subsided within 1-month, local discomfort subsided within 4 months. Remained asymptomatic for 1 year post-explantation. **Case 2:** subjectively feeling better within a few days post-explantation, all symptoms gone within 1 month from explantation surgery. **Case 3:** after 3 weeks, photophobia had disappeared and chronic fatigue had subsided. Normal energy after 6 weeks. **Case 4:** within 1 week, symptoms had improved, within 2 weeks from surgery patient reported complete absence of symptoms she had experienced for 10 years. Six months post-surgery she remained asymptomatic. **Case 5:** three days post-explantation showed resolution of the chronic diarrhoea. Within 1 month, the fatigue had eased significantly, and hot flashes, insomnia, and irregular periods had resolved. **Case 6:** four weeks post-surgery, fatigue had resolved and foot pain had lessened. Within 3 months, symptoms had entirely resolved. **Case 7:** one month post-explantation, fatigue and discomfort had disappeared. No information given on memory impairment.

BIA-ALCL is a rare form of non-Hodgkin’s lymphoma that has recently garnered consideration as a unique clinicopathologic entity by the World Health Organization [18, 19]. The most common clinical presentation of BIA-ALCL is a peri-implant effusion seen within 551 cases out of a total 1130 (49%) as of January 2022 [20]. The pathogenesis of BIA-ALCL is unclear though chronic inflammation by the presence of biofilm results in persistent T-cell transformation and lymphomagenesis [13, 19]. Although rare, it is an important diagnosis to exclude in a patient presenting with breast capsular contracture and peri-implant effusions. Breast US remains the gold-standard for detecting implant rupture, seroma or any peri-implant or capsular mass [21–23].

The main treatment for breast implant infections includes en bloc explantation, explant with total capsulectomy, explant with partial capsulectomy or open capsulotomy with long-term surveillance. The contamination is usually during surgery, and in revision surgery, handling of an implant through aseptic techniques and funnelling is an important practice change.

Our cases highlight the importance of identifying a common complication of breast implants in the form of capsular contracture and the associated bacterial infection of *C. acnes*. Surgeons may have a high degree of suspicious of capsular contractures with BII; however, the symptoms of capsular contracture can present at different points post-surgery. The foundation of a triple assessment and workup including broader differentials such as common pathogens should be part of the armamentarium to recognise skin-related pathogens and practice of implantation.

## CONCLUSION

*Cutibacterium acnes* is an important under recognised bacterium that plays a role in BII. Surgeons should be aware of this potential under recognised complication and have an open discussion with patients before breast surgery including potential risks and management. Currently, revision surgery with explant and capsulectomy is the only available treatment option for women with severe capsular contracture.

## AUTHORS’ CONTRIBUTIONS

The authors contributed to the conception and design of the manuscript, revised it critically for important intellectual content, approved the final version to be published and agreed to be accountable for all aspects of the work.

## Data Availability

The data are deemed confidential and under ethics cannot be disseminated openly because of confidentiality and privacy.

## References

[1] Plastic Surgery Statistics Report . ASPS National Clearinghouse of Plastic Surgery Procedural Statistics. Arlington Heights, IL, USA: American Society of Plastic Surgeons. 2020.

[2] Hopper I, Parker E, Pellegrini B, et al. The Australian Breast Device Registry 2016 Report. Melbourne: Monash University, Department of Epidemiology and Preventive Medicine, 2018.

[3] Tang SY, Israel JS, Afifi AM. Breast implant illness: symptoms, patient concerns, and the power of social media. Plast Reconstr Surg 2017;140:765e–6e. 28753149 10.1097/PRS.0000000000003785

[4] Washer LL, Gutowski K. Breast implant infections. Infect Dis Clin North Am 2012;26:111–25. 22284379 10.1016/j.idc.2011.09.003

[5] Hart D . Overcoming complications of breast implants. Plast Surg Nurs 2003;23:72. 10.1097/00006527-200323020-00005 14533571

[6] Araco A, Caruso R, Araco F, Overton J, Gravante G. Capsular contractures: a systematic review. Plast Reconstr Surg 2009;124:1808–19. 19952637 10.1097/PRS.0b013e3181bf7f26

[7] Headon H, Kasem A, Mokbel K. Capsular contracture after breast augmentation: an update for clinical practice. Arch Plast Surg 2015;42:532–43. 26430623 10.5999/aps.2015.42.5.532 PMC4579163

[8] Bhatia, A., Maisonneuve, J. F., & Persing, D. H. *Propionibacterium acnes* and chronic diseases. In: Knobler SL, et al. eds. The Infectious Etiology of Chronic Diseases: Defining the Relationship, Enhancing the Research, and Mitigating the Effects: Workshop Summary. 2004, Washington, DC, USA: National Academies Press. 74–80. 22379643

[9] Hsu JE, Bumgarner RE, Matsen FA III. Propionibacterium in shoulder arthroplasty: what we think we know today. JBJS 2016;98:597–606. 10.2106/JBJS.15.00568 27053589

[10] Ahn CY, Ko CY, Wagar EA, Wong RS, Shaw WW. Microbial evaluation: 139 implants removed from symptomatic patients. Plast Reconstr Surg 1996;98:1225–9. 8942908 10.1097/00006534-199612000-00016

[11] Portillo ME, Corvec S, Borens O, Trampuz A. *Propionibacterium acnes*: an underestimated pathogen in implant-associated infections. Biomed Res Int 2013;2013:804391. 24308006 10.1155/2013/804391 PMC3838805

[12] Cove JH, Holland KT, Cunliffe WJ. Effects of oxygen concentration on biomass production, maximum specific growth rate and extracellular enzyme production by three species of cutaneous propionibacteria grown in continuous culture. Microbiology 1983;129:3327–34. 10.1099/00221287-129-11-3327 6663280

[13] Castellano M, Marín M, Alcalá L, Cunnas I, Rodríguez B, Ruíz MJ, et al. Exhaustive diagnosis of breast implants with capsular contracture: the microbiology laboratory as a major support. J Plast Reconstr Aesthet Surg 2022;75:3085–93. 10.1016/j.bjps.2022.06.014 35872019

[14] Flemming HC, Wingender J, Szewzyk U, Steinberg P, Rice SA, Kjelleberg S. Biofilms: an emergent form of bacterial life. Nat Rev Microbiol 2016;14:563–75. 27510863 10.1038/nrmicro.2016.94

[15] De la Fuente-Núñez C, Reffuveille F, Fernández L, Hancock RE. Bacterial biofilm development as a multicellular adaptation: antibiotic resistance and new therapeutic strategies. Curr Opin Microbiol 2013;16:580–9. 23880136 10.1016/j.mib.2013.06.013

[16] Coenye T, Peeters E, Nelis HJ. Biofilm formation by *Propionibacterium acnes* is associated with increased resistance to antimicrobial agents and increased production of putative virulence factors. Res Microbiol 2007;158:386–92. 17399956 10.1016/j.resmic.2007.02.001

[17] Pittet B, Montandon D, Pittet D. Infection in breast implants. Lancet Infect Dis 2005;5:94–106. 15680779 10.1016/S1473-3099(05)01281-8

[18] de Boer M, van Leeuwen FE, Hauptmann M, Overbeek LI, de Boer JP, Hijmering NJ, et al. Breast implants and the risk of anaplastic large-cell lymphoma in the breast. JAMA Oncol 2018;4:335–41. 29302687 10.1001/jamaoncol.2017.4510 PMC5885827

[19] Quesada AE, Medeiros LJ, Clemens MW, Ferrufino-Schmidt MC, Pina-Oviedo S, Miranda RN. Breast implant-associated anaplastic large cell lymphoma: a review. Mod Pathol 2019;32:166–88. 30206414 10.1038/s41379-018-0134-3

[20] Center for Devices and Radiological Health , US Food and Drug Administration: FDA Update on the Safety of Silicone Gel-Filled Breast Implants. https://www.fda.gov/media/80685/download (8 October 2022, date last accessed).

[21] Kaderbhai A, Broomfield A, Cuss A, Shaw K, Deva AK. Breast implants: a guide for general practice. Aust J Gen Pract 2021;50:484–90. 34189547 10.31128/AJGP-01-20-5188

[22] Ramamurthi A, Patel H, Srinivasa DR. Breast implant-associated anaplastic large cell lymphoma: a Google Trends analysis. Aesthetic Plast Surg 2022;46:1653–61. 35441237 10.1007/s00266-022-02877-9

[23] Adrada BE et al. Breast implant associated anaplastic large cell lymphoma: sensitivity, specificity, and findings of imaging studies in 44 patients. Breast Cancer Res Treat 2014;147:1–14. 25073777 10.1007/s10549-014-3034-3

[24] McCarthy PH, Teitler NA, Hon HH, Miller JJ. Breast implant illness and *Cutibacterium acnes*: a case report. Plast Reconstr Surg Glob Open 2022;10:e4146. 35242491 10.1097/GOX.0000000000004146 PMC8884533

[25] Metzinger SE, Homsy C, Chun MJ, Metzinger RC. Breast implant illness: treatment using total capsulectomy and implant removal. Eplasty 2022;22:e5. 35602522 PMC9097901

[26] McGuire P, Glicksman C, Wixtrom R, James Sung C, Hamilton R, Lawrence M, et al. Microbes, histology, blood analysis, enterotoxins, and cytokines: findings from the ASERF systemic symptoms in women—biospecimen analysis study: part 3. Aesthet Surg J 2022;43:230–244. 10.1093/asj/sjac225 PMC9896138 35980942

[27] Katsnelson JY, Spaniol JR, Buinewicz JC, Ramsey FV, Buinewicz BR. Outcomes of implant removal and capsulectomy for breast implant illness in 248 patients. Plast Reconstr Surg Glob Open 2021;9:e3813. 34513545 10.1097/GOX.0000000000003813 PMC8423394

[28] Lee M, Ponraja G, McLeod K, Chong S. Breast implant illness: a biofilm hypothesis. Plast Reconstr Surg Glob Open 2020;8:e2755. 32440423 10.1097/GOX.0000000000002755 PMC7209857

[29] Dowden RV . Periprosthetic bacteria and the breast implant patient with systemic symptoms. Plast Reconstr Surg 1994;94:300–5. 8041821 10.1097/00006534-199408000-00013

